# Evaluation of Sutureless, Glueless, Flapless, Intrascleral Fixated Posterior Chamber Intraocular Lens in Children with Ectopia Lentis

**DOI:** 10.1155/2018/3212740

**Published:** 2018-08-29

**Authors:** Kannan NB, Piyush Kohli, Bhanu Pratap Singh Pangtey, Kim Ramasamy

**Affiliations:** Department of Vitreo-Retinal Services, Aravind Eye Hospital and Post Graduate Institute of Ophthalmology, 1 Anna Nagar, Madurai, Tamil Nadu 625020, India

## Abstract

**Aim:**

This paper aims at evaluating refractive outcome and complication profile of sutureless, glueless, flapless, intrascleral fixation of intraocular lens (SFIOL) in pediatric population.

**Methods:**

This retrospective study included patients ≤18 years of age who underwent SFIOL for ectopia lentis. Details obtained included preoperative uncorrected visual acuity (UCVA), cycloplegic refraction, and best-corrected visual acuity (BCVA); intraoperative complications; and postoperative UCVA, cycloplegic refraction, and BCVA and complications.

**Results:**

Median pre- and postoperative UCVA was logMAR 1.78 (Snellen 20/1200) and logMAR 0.30 (Snellen 20/40), respectively, (*p* < 0.001). Median pre- and postoperative BCVA was logMAR 0.24 (Snellen 20/34) and logMAR 0.18 (Snellen 20/30), respectively. UCVA ≥20/60 was attained in 90% of eyes. BCVA ≥20/30 was attained in 85.0% of eyes. Most common early postoperative complications were hyphaema (10%), transient vitreous hemorrhage (2.5%), and ocular hypotony (2.5%). None of these developed any long-term sequelae. Only one case of subluxation of IOL was seen. No case of late endophthalmitis or retinal detachment was seen.

**Conclusion:**

Since refractive error induced is minimal, the procedure is suitable for IOL implantation in children, who are noncompliant with spectacles. The complication profile is similar to that reported in adults.

## 1. Introduction

Optical rehabilitation of childhood aphakia, in the presence of an unstable capsule-zonule complex, is one of the most challenging tasks faced by the ophthalmologists. Numerous techniques like anterior chamber IOL (ACIOL), iris-fixated IOL (IFIOL), and scleral fixated IOL (SFIOL) have been developed for such eyes [1–8]. Long-term complications associated with ACIOL and IFIOL like corneal decompensation due to endothelial cell loss; chronic uveitis leading to peripheral anterior synechiae and glaucoma; and iris chaffing and pupillary constriction make them an unsafe option for children, due to their long-life expectancy [1–10].

By the virtue of its near physiological anatomical location, such complications are rare in the case of SFIOL [6, 7, 9–13]. However, suspending the SFIOL with the help of 10-0 polypropylene suture is associated with a number of suture-related complications like suture erosion, suture knot exposure, and recurrent dislocation(s) due to dissolution of the suture with time [1–16]. To overcome the suture-related complications, various techniques of sutureless scleral fixation of IOL have been described.

The results of sutureless SFIOL have been encouraging in adult eyes [17–23]. However, the efficacy and safety of this technique in the pediatric age group has not been adequately studied. This study was undertaken to evaluate the refractive outcome and complication profile of sutureless, glueless, flapless, intrascleral fixation of PCIOLs in pediatric patient presenting with congenital ectopia lentis.

## 2. Materials and Methods

This was a retrospective study conducted at Aravind Eye Hospital, Madurai, India. Case records of patients under 18 years of age who underwent SFIOL for congenital ectopia lentis from January 2012 to December 2016 were analyzed.

Preoperative evaluation included uncorrected visual acuity (UCVA), best-corrected visual acuity (BCVA), cycloplegic refraction, intraocular pressure, and a comprehensive anterior and posterior segment evaluation to find any pathological cause of decreased BCVA. Any intraoperative and postoperative complication(s), along with their management, were noted. At each follow-up, the examination included UCVA, BCVA, cycloplegic refraction, intraocular pressure, and a comprehensive anterior and posterior segment evaluation to see for any postoperative complications. The intra- and postoperative complication(s) were managed as per the universally accepted protocols. Causes of poor postoperative BCVA were discerned in all cases.

### 2.1. Surgical Technique

All surgeries were done under general anesthesia by a single surgeon (Dr. NB). Superior 270-degree conjunctival peritomy is followed by light scleral cautery to achieve adequate hemostasis. Two partial-thickness scleral pockets are then made, with the help of 25-gauge (G) microvitreoretinal (MVR) trocar blade, for permanent incarceration of the IOL haptics. These pockets are created 180-degree apart, parallel to the limbus, and 1.5–2 mm away from the limbus. A scleral tunnel is made superiorly with the help of a crescent blade. Next, three standard 25G pars plana vitrectomy (PPV) sclerostomy ports are constructed at 2, 10, and 4′o clock. Then, two ciliary sulcus-based sclerotomies are created close to the scleral pockets for externalization of IOL haptics, using a 24G needle. First, lensectomy and anterior vitrectomy are performed.

Entry into the anterior chamber is then made through the scleral tunnel with the help of a keratome blade. A standard three-piece nonfoldable IOL (Aurolab, Madurai, India) is implanted. With the tailing haptic fixated at the scleral incision, the leading haptic is grasped at the tip with a 25-gauge end-gripping forceps (Alcon Laboratories, Fort Worth, Texas, USA) and pulled out through the sclerotomy. The haptic is then tucked into the scleral pocket. Then, the tailing haptic is inserted into the posterior chamber. The IOL that does not fall as one haptic is already incarcerated in the scleral pocket. The second haptic is visualized with the help of a binocular indirect ophthalmomicroscope (BIOM), grasped, and pulled out through the second sclerotomy. The tailing haptic is also tucked into the limbus-parallel scleral pocket. Finally, centralization of the IOL is done by adjusting the amount of haptic in each pocket. The vitrectomy ports, the sclera tunnel, and the conjunctiva are finally sutured. All the sites were inspected for wound leakage.

In case IOL subluxation is seen in the postoperative period, it can be centralized easily by performing a localized peritomy over the 2 scleral pockets and adjusting the length of haptic tucked in the scleral pocket.

### 2.2. Statistics

Statistical analysis was performed with Stata statistical software, version 11.1 (StataCorp, College Station, Texas, USA). Continuous variables were expressed as mean (±standard deviation), and categorical variables were expressed as percentages. Any association between categorical data was seen with help of the chi-square/Fisher exact test, while difference in continuous data between 2 groups was seen with help of the Student *t*-test/Mann–Whitney *U* test. Change in variables after a procedure was done with the help of the paired *t*-test. *P* value less than 0.05 was considered to be statistically significant.

## 3. Results

**Figure 1:**
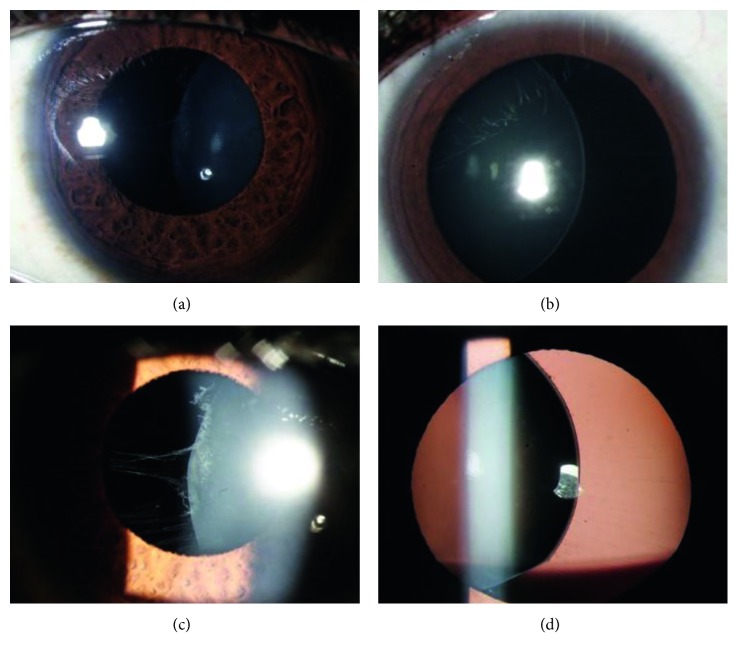
Preoperative images of patients with ectopia lentis.

The study included 40 eyes of 25 patients (12 males; 13 females) with mean age of 13.4 ± 3.7 years (range, 6–18 years).  Figure 1 shows preoperative images of ectopia lentis. Median preoperative and postoperative UCVA was logMAR 1.78 (Snellen 20/1200) and logMAR 0.24 (Snellen 20/34), respectively. Median preoperative and postoperative BCVA was logMAR 0.30 (Snellen 20/40) and logMAR 0.18 (Snellen 20/30), respectively. However, 90% of eyes (*n* = 36/40) attained UCVA ≥20/60, and 67.5% (*n* = 27/40) attained UCVA ≥20/40. Improvement in BCVA by at least 2 Snellen line was achieved in 47.5% (*n* = 19/40) of eyes. BCVA ≥20/30 was attained in 34 eyes (85.0%) (Table 1).

Postoperative emmetropia was attained in 32.5% (*n* = 13/40) of eyes. Average spherical equivalent (SE) was 0.26 ± 0.97 DS, while average cylindrical correction was 0.68 ± 0.72 DC. SE was ≤1 DS in 88.2% of eyes (*n* = 33/40), while cylindrical correction was ≤1 DC in 72.5% of eyes (*n* = 29/40). While 47.5% (*n* = 19/40) of eyes had no astigmatism, only 12.5% (*n* = 5/40) of eyes had astigmatism ≥1.5 DC.

There were no intraoperative complications. Most common early postoperative complications were hyphaema (*n* = 4, 10.0%), transient vitreous hemorrhage (*n* = 1, 2.5%), and ocular hypotony (*n* = 1, 2.5%) (Table 2). However, there was no choroidal effusion. Good corneal clarity was obtained in immediate postoperative follow-up in all the cases. Two eyes had elevated intraocular pressure (>30 mm Hg) prior to surgery, which came to normal after the surgery. One patient developed inferior subluxation of IOL and had to be refixated. None of these eyes developed long-term sequelae complications like glaucoma, cystoid macular edema, bullous keratopathy, or endophthalmitis.

**Figure 2:**
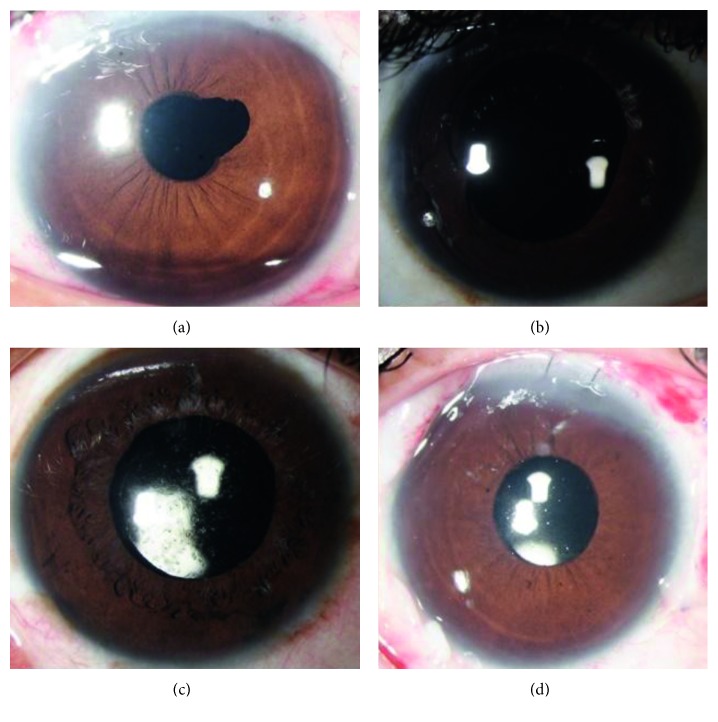
Postoperative images of patients after sutureless, glueless, flapless, intrascleral fixated intraocular lens.

The median follow-up of the patients was 12 months (range, 12–62 months). The IOL was well centered in all the eyes at the last follow-up and required no active intervention (Figure 2).

## 4. Discussion

Scleral fixation of IOL in the posterior chamber is the current procedure of choice for surgical rehabilitation of aphakia in the absence of adequate capsular support [1–8]. However, the conventional method of suturing the IOL is associated with a number of side effects related to the suture and extensive IOL manipulation in the anterior chamber [1–10].

The purpose of visual rehabilitation by IOL implantation is to achieve minimum refractive error. The average astigmatism induced in our study was 0.68 ± 0.77 DC, which is a small value. Nearly half of the eyes were astigmatically neutral while around three-fourth of the eyes had astigmatism less than 1 DC. Around 90% of eyes attained UCVA ≥20/60 and had spherical equivalent less than 1 DS. Since refractive error induced in our study was minimal, the procedure is suitable for implantation of IOL in children who tend to be noncompliant with their spectacles. With good uncorrected vision, chances of patients developing amblyopia in the postoperative period reduce.

The technique used in this study for SFIOL was originally described by Gabor and Pavlidis [20] has multiple intraoperative advantages over the conventional suturing technique. The conventional technique is associated with globe collapse and hypotony, especially during the passage of scleral sutures. The anterior chamber instability can lead to corneal endothelial damage as well as compromise the exact point of needle egress [11, 24]. This discrepancy in placement of scleral suture, either due to the distance from limbus or angular position, can cause IOL decentration and tilt [25]. However, the technique used in this study ensures greater globe stability during the surgery. This is because a majority of manipulations were made in the vitreous cavity. No case of bullous keratopathy or clinically significant IOL tilt was seen in our study patients. This technique also ensured easy intraoperative IOL centration by adjusting the length of haptic incarceration. As maneuvers performed are reduced, risk for intraoperative trauma also reduces.

The postoperative complications associated with the sutureless technique were also less than the conventional technique. No apparent IOL tilt was noticed in any of the patients in our study patients as the haptics were placed in the intrascleral tunnels without any traction. None of the eyes had severe inflammation in the form of fibrin since minimum intraocular manipulation was done during IOL fixation. Reduced postoperative inflammation made the visual rehabilitation faster. Since the tips of the haptics were buried intrasclerally, conjunctival erosion was not seen in any of the eyes. This also reduced the chances of late-onset endophthalmitis as suture erosion can serve as a direct communication channel between the outside environment and the eye [9].

This technique had several advantages over the glued sutureless SFIOL also. Firstly, the scleral tunnels have been demonstrated, using anterior segment ultrasound, to achieve a leak-free closure even without the application of glue [21]. Avoiding the use of glue not only reduces the cost of surgery, but also avoids the theoretic chance of transmission of infectious agents associated with the use of glue [9, 18]. Even in our study, no case of postoperative hypotony or choroidal detachment was seen in our study. Secondly, easier refixation of IOL is possible in the case of postoperative subluxation, by just adjusting the length of haptic incarcerated. The technique avoids the need of reopening flaps, which is technically difficult due to fibrosis.

This study is the largest series evaluating the safety and efficacy of sutureless, glueless, intrascleral fixation in the pediatric population. The technique has multiple advantages including reduction in cost of surgery and surgical time, as well as intra and postoperative complications. Also, resurgery is relatively easier. Easy centration of IOL can allow the use of multifocal and toric IOLs. The disadvantages of the technique include its learning curve and requirement for specialized instruments. Limitations of the study include its retrospective nature and short duration of follow-up.

## Figures and Tables

**Table 1:** Preoperative and postoperative uncorrected visual acuity (UCVA) and best-corrective visual acuity (BCVA) of the patients.

	Preoperative	Postoperative
UCVA	LogMAR 1.78 (range, logMAR 1.08–1.78)	LogMAR 0.30 (range, logMAR 0.00–1.00)
	Snellen 20/1200	Snellen 20/40
BCVA	LogMAR 0.24 (range, logMAR 0.00–1.18)	LogMAR 0.18 (range, logMAR 0.00–0.48)
	Snellen 20/34	Snellen 20/30
Eye with UCVA ≥20/60	0	90.0% (*n* = 36/40)
Eyes with BCVA ≥20/30	50.0% (*n* = 20/40)	85.0% (*n* = 34/40)
2-line gain in BCVA	NA	47.5% (19/40)

**Table 2:** Postoperative complications in each group.

S.No.	Name of complication	Number of eyes	Percentage
Early postoperative complications			
1	Hyphaema	4	10.0
2	Transient vitreous hemorrhage	1	2.5
3	IOL capture	1	2.5
4	Postoperative hypotony	1	2.5
5	Choroidal effusion	0	0
6	Fibrin reaction	0	0
7	Endophthalmitis	0	0

Late postoperative complications			
1	IOL subluxation	1	2.5
2	Glaucoma	0	0
3	Recurrent uveitis	0	0
4	Posterior synechiae (>2 clock hour)	0	0
5	Cystoid macular edema	0	0
6	Bullous keratopathy	0	0
7	Retinal detachment	0	0

## Supplementary Materials

Supplementary Materials Raw data for “Evaluation of sutureless, glueless, flapless, intrascleral fixated posterior chamber intraocular lens in children with ectopia lentis.” Description of data: (1) Age at time of SFIOL: age in years at the time of scleral fixated intraocular surgery; (2) Sex: M is male and F is female; (3) Pre-UV (S) means preoperative uncorrected visual acuity in Snellen; (4) Pre-UV (L) means preoperative uncorrected visual acuity in logMAR; (5) Pre-BV (S) means preoperative best-corrected visual acuity in Snellen; (6) Pre-BV (L) means preoperative best-corrected visual acuity in logMAR; (7) Postop Cx: any postoperative complication; (8) Post-UV (S) means postoperative uncorrected visual acuity in Snellen; (9) Post-UV (L) means postoperative uncorrected visual acuity in logMAR; (10) Post R (S) means postoperative refractive spherical error; (11) Post R (C) means postoperative refractive cylindrical error; (12) Emet means postoperative emmetropia: 0 means no and 1 means yes; (13) <1 DC means postoperative refractive cylindrical error <1 DC; (14) Seq means spherical equivalent; (15) Post-BV (S) means postoperative best-corrected visual acuity in Snellen; (16) Post-BV (L) means postoperative best-corrected visual acuity in logMAR; (17) FU means total follow-up of the patient; (18) 2-line means 2-line improvement in best-corrected visual acuity after the surgery: 0 means no and 1 means yes.
